# Sulfur Stable Isotope Discrimination in Rice: A Sulfur Isotope Mass Balance Study

**DOI:** 10.3389/fpls.2022.837517

**Published:** 2022-03-10

**Authors:** Viviana Cavallaro, Moez Maghrebi, Mariachiara Caschetto, Gian Attilio Sacchi, Fabio Francesco Nocito

**Affiliations:** ^1^Dipartimento di Scienze Agrarie e Ambientali—Produzione, Territorio, Agroenergia, Università degli Studi di Milano, Milan, Italy; ^2^Dipartimento di Scienze della Vita e Biologia dei Sistemi, Università degli Studi di Torino, Turin, Italy; ^3^Dipartimento di Scienze dell’Ambiente e della Terra, Università degli Studi di Milano-Bicocca, Milan, Italy

**Keywords:** fractionation, *Oryza sativa* L., sulfate uptake, sulfur assimilation, sulfur stable isotopes

## Abstract

The use of sulfur (S) stable isotopes to study S metabolism in plants is still limited by the relatively small number of studies. It is generally accepted that less S stable isotope discrimination occurs during sulfate (SO_4_^2–^) uptake. However, S metabolism and allocation are expected to produce separations of S stable isotopes among the different plant S pools and organs. In this study, we measured the S isotope composition of the main S pools of rice plants grown under different SO_4_^2–^ availabilities in appropriate closed and open hydroponic-plant systems. The main results indicate that fractionation against ^34^S occurred during SO_4_^2–^ uptake. Fractionation was dependent on the amount of residual SO_4_^2–^ in the solution, showing a biphasic behavior related to the relative expression of two SO_4_^2–^ transporter genes (*OsSULTR1;1* and *OsSULTR1;2*) in the roots. S isotope separations among S pools and organs were also observed as the result of substantial S isotope fractionations and mixing effects occurring during SO_4_^2–^ assimilation and plant S partitioning. Since the S stable isotope separations conserve the memory of the physiological and metabolic activities that determined them, we here underline the potential of the ^32^S/^34^S analysis for the detailed characterization of the metabolic and molecular processes involved in plant S nutrition and homeostasis.

## Introduction

Since 1865, sulfur (S) has been recognized as an essential element for plant growth (Sachs, 1865; Epstein, 2000). In plants, S is found in the amino acid cysteine and methionine, short peptides, vitamins and cofactors, and secondary compounds (Takahashi et al., 2011).

Plants mainly utilize sulfate (SO_4_^2–^), an inorganic form of oxidized S present in the soil solution, to support their growth. SO_4_^2–^ is taken up by roots and allocated to various sink tissues, where it is stored in the cell vacuoles or assimilated into S organic (S_org_) compounds (Saito, 2004; Takahashi et al., 2011). To accomplish the assimilation of S into biomolecules, SO_4_^2–^ is first activated by ATP sulfurylase to adenosine-5′-phosphosulfate (APS), which is then channeled toward reduction or sulfation (Leustek et al., 2000). Most of the APS enters the reductive pathway along which sulfite and, subsequently, sulfide are produced through two sequential reactions catalyzed by APS reductase and sulfite reductase, respectively. Sulfide is finally incorporated into *O*-acetylserine (OAS) to form cysteine in a reaction catalyzed by OAS(thiol)lyase (Takahashi et al., 2011). In the sulfation pathway, the APS is first phosphorylated by APS kinase to form 3′-phosphoadenosine-5′-phosphosulfate, the donor of SO_4_^2–^ groups for a variety of sulfation reactions catalyzed by sulfotransferases (Günal et al., 2019).

Sulfur has four stable isotopes, namely, ^32^S, ^33^S, ^34^S, and ^36^S; their relative abundances are 0.9499, 0.0075, 0.0425, and 0.0001 atom fraction, respectively (De Laeter et al., 2003). Mass differences between the S isotopes result in small but significant variations in their chemical and physical properties, which may produce considerable separation of the S isotopes during chemical reactions. The most abundant isotopes, i.e., ^32^S and ^34^S, are now commonly measured using elemental analyzers coupled with Isotope Ratio Mass Spectrometers (IRMS), and S isotope abundance is generally reported in δ notation (δ^34^S) as parts per thousand (‰) deviation relative to the Vienna-Cañon Diablo Troilite (VCDT; Coplen and Krouse, 1998) standard as follows:


$$\delta^{34}\,S\,\left(\text{‰}\right)=\frac{R_{s\,a\,m\,p\,l\,e}-R_{s\,t\,a\,n\,d\,a\,r\,d}}{R_{s\,t\,a\,n\,d\,a\,r\,d}}\cdot 1,000$$

where *R*_*sample*_ and *R*_*standard*_ are the isotope ratios (^34^S/^32^S) of the sample and standard, respectively.

Unlike what has happened with carbon and nitrogen, the natural abundance S stable isotope analysis techniques have so far scarcely been employed to study S allocation and metabolism in plants (Trust and Fry, 1992; Tcherkez and Tea, 2013), mainly due to the lack of knowledge about the ^32^S/^34^S isotope effects occurring during S metabolism and partitioning among the different organs. Most of the irreversible reactions involving S discriminate between ^32^S and ^34^S by favoring the lighter ^32^S isotope, thus enriching in ^34^S the residual substrate molecules left behind. That is to say that irreversible reactions that do not consume all the substrate may likely produce a detectable separation of the S stable isotopes, i.e., a fractionation, at natural abundance, providing crucial insights into the understanding of S metabolic fluxes inside the plants, without the need for costly labeling experiments with radioactive (^35^S) or stable (^34^S) isotopes (Tcherkez and Tea, 2013).

Sulfate uptake and allocation in plants involve a family of SO_4_^2–^ transporter proteins whose activities are tightly regulated and coordinated with those of the assimilation pathways to control plant S homeostasis (Buchner et al., 2004; Gigolashvili and Kopriva, 2014; Sacchi and Nocito, 2019; Takahashi, 2019). A few pioneering studies indicated that a less S isotope discrimination occurs during SO_4_^2–^ uptake since the isotope composition measured for plant total S (S_tot_) is typically depleted in ^34^S by 1–2‰ with respect to that measured for the SO_4_^2–^ source feeding the plants (Mekhtiyeva, 1971; Krouse et al., 1991). In contrast, less is known about the S isotope composition of the SO_4_^2–^ ions in the plant tissues, which should reflect the metabolic activities in which SO_4_^2–^ is involved as a substrate. Although the isotope effects linked to SO_4_^2–^ metabolism largely remain to be investigated in plants, it is possible to suppose that reductive SO_4_^2–^ assimilation fractionates against ^34^S, since it involves changes in the covalent bonding of the S atoms (Rees, 1973). Significant isotope effects have been reported for bacterial SO_4_^2–^ reduction, which enriches both the sulfide produced in the lighter ^32^S isotope and the remaining SO_4_^2–^ in the heavier ^34^S isotope (Thode et al., 1949; Kemp and Thode, 1968).

This study presents a detailed study on the dynamics of S stable isotopes occurring in appropriate closed or steady-state hydroponic-plant systems to dissect the ^32^S/^34^S isotope effects associated with SO_4_^2–^ uptake, allocation, and metabolism in rice plants. In this study, we also provided the first complete S isotope mass balance in rice which considers organic and inorganic S pools in roots and shoots.

## Materials and Methods

### Plant Material and Pre-growing Conditions

Rice (*Oryza sativa* L. cv. Vialone Nano) caryopses were surface sterilized with 70% (v:v) ethanol for 1 min, washed three times with sterile deionized water, and finally sown on filter paper saturated with deionized water to be incubated in the dark at 26°C. After 7 days, seedlings selected for uniform growth were transferred into 3-L plastic tanks (18 seedlings per tank), containing the following complete nutrient solution: 1.5 mM KNO_3_, 1 mM Ca(NO_3_)_2_, 100 μM MgSO_4_, 250 μM NH_4_H_2_PO_4_, 25 μM Fe-EDTA, 46 μM H_3_BO_3_, 9 μM MnCl_2_, 1 μM ZnCl_2_, 0.3 μM CuCl_2_, 0.1 μM (NH_4_)_6_Mo_7_O_24_, and 30 μM Na_2_O_3_Si (pH 6.5). Seedlings were kept for a 14-days pre-growing period in a growth chamber maintained at 26°C and 80% relative humidity during the 16-h light period and at 22°C and 70% relative humidity during the 8-h dark period. The photosynthetic photon flux density was 400 μmol m^–2^ s^–1^. Nutrient solutions were renewed two times a week to minimize nutrient depletion. At the end of the pre-growing period, roots were gently washed for 30 min in 3 L of deionized water (>18.2 MΩ cm). Plants were then transferred into fresh solutions and used in two distinct experimental setups (A and B). The parts of the plants were sampled, frozen in liquid N_2_, and stored at −80°C for further analysis.

### Experimental Setup and Tissue Sampling

In experimental setup A, pre-grown rice plants were transferred into fresh complete nutrient solutions and then grown further, under the same conditions described before, for 3–11 days, not renewing the growing media. Both plants and nutrient solutions were sampled at the beginning of the experiment and every day (starting from the third day).

In experimental setup B, pre-grown rice plants were transferred into fresh complete nutrient solutions (+SO_4_^2–^) or fresh minus sulfate nutrient solutions (−SO_4_^2–^), in which an equimolar amount of MgCl_2_ replaced MgSO_4_. Plants were grown under these conditions for 48 or 72 h by renewing the growing media every day.

In both the experimental setups, before sampling, plant roots were washed for 30 min in 3 L of deionized water to remove SO_4_^2–^, which was not absorbed, from the root apoplast. After washing, plants were gently blotted with paper towels, shoots were separated from roots, and then, both were frozen in liquid N_2_ and stored at −80°C for further analysis.

### Xylem Sap Sampling

In each sampling period (experimental setup B in the presence of SO_4_^2–^), the shoots of four rice plants were cut at 1 cm above the roots with a microtome blade to collect, with a micropipette, the xylem sap exuded from the lower cut surface during a 90-min period (Maghrebi et al., 2021).

### Preparation of Samples for Sulfur Isotope Analysis and Quantitative Determination of the Sulfur Pools

Frozen samples were ground to a fine powder using mortar and pestle in liquid N_2_ and stored frozen in a cryogenic tank.

For S_tot_ analysis, powder samples of 5 g [fresh weight (FW)] were digested at 150°C in 10 ml 2:1 (v:v) nitric:perchloric acid mixture, in order to quantitatively convert all the S forms into SO_4_^2–^ (Blair and Till, 2003). Samples were then added with 1 ml of concentrated HCl and finally evaporated to dryness at 200°C to release any HNO_3_ still present. The mineralized material was dissolved in 50 ml of deionized water and then brought to pH 2.0 with a tiny volume of 6 N HCl.

Sulfate was extracted from roots and shoots by homogenizing powder samples of 5 g (FW) in 50 ml of deionized water. After heating at 80°C for 40 min, the extracts were filtered and then brought to pH 2.0 with a tiny volume of 6 N HCl.

Residual nutrient solutions were boiled to evaporate water until their volumes were reduced to 50 ml. Samples were then filtered and brought to pH 2.0 with 6 N HCl.

Xylem sap samples were diluted with deionized water to a final volume of 50 ml, filtered, and then brought to pH 2.0 with a tiny volume of 6 N HCl.

Aliquots of 2 ml were collected from each diluted sample for the quantitative determination of SO_4_^2–^, using the turbidimetric method described by Tabatabai and Bremner (1970). The SO_4_^2–^ ions of each sample were precipitated overnight as BaSO_4_ by adding 2.5 ml of a 0.5 M BaCl_2_ solution. BaSO_4_ was then collected by centrifugation, washed two times in 2 ml of deionized water, dried at 80°C, ground to a fine powder, and finally used for the S isotope analyses.

The amount of the S_org_ pool of both root and shoot extracts was estimated as follows:


$${\text{S}}_{\text{org}}={\text{S}}_{\text{tot}}-{\text{SO}}_{4}^{2-}$$

### Sulfur Isotope Analysis

The δ^34^S values of samples were measured using a Flash 2000 HT elemental analyzer coupled, *via* a ConFLo IV Interface, with a Delta V Advantage IRMS and interconnected to the software Isodat 3.0 (Thermo). The reaction tube, packed with tungstic oxide and copper wires separated by Quartz wool, was maintained at 1,020°C. The He carrier gas flow was 150 ml min^–1^. The O_2_ purge for flash combustion was 3 s at a flow rate of 250 ml min^–1^ per sample. The temperature of the gas chromatography separation column was 90^°^C. The SO_2_ reference gas pulse was introduced three times (20 s each) at the beginning of each run.

Samples (BaSO_4_ precipitates and reference materials) were weighed in tin capsules. Capsules were carefully closed by folding them with cleaned tweezers and then transferred to the autosampler. The run time of the analysis was approximately 500 s for a single run. The analysis of each sample was performed five times. Calibration was performed using three secondary reference materials provided by the International Atomic Energy Agency (IAEA): IAEA-S-1 (δ^34^S = −0.30 ± 0.03‰); IAEA-S-2 (δ^34^S = 22.62 ± 0.08‰); IAEA-S-3 (δ^34^S = −32.49 ± 0.08‰). Two in-house standards were used for normalization and analytical quality assurance.

The data are reported in δ^34^S notation, which is standardized to the VCDT international reference scale as follows:


$$\delta^{34}\,S\,\left(\text{‰}\right)=\frac{R_{s\,a\,m\,p\,l\,e}-R_{s\,t\,a\,n\,d\,a\,r\,d}}{R_{s\,t\,a\,n\,d\,a\,r\,d}}\cdot 1,000$$

The mass spectrometric uncertainty (1 σ) on the individual δ^34^S measurements was better than 0.05‰.

The δ^34^S values of the S_org_ pool were estimated by imposing the following mass balance:


$$\delta^{34}\,{\text{S\_S}}_{\text{tot}}\cdot {\text{S}}_{\text{tot}}=\left(\delta^{34}\,{\text{S\_SO}}_{4}^{2-}\cdot {\text{SO}}_{4}^{2-}\right)+\left(\delta^{34}\,\text{S}\,\_\,{\text{S}}_{\text{org}}\cdot {\text{S}}_{\text{org}}\right)$$

where SO_4_^2–^ and S_tot_ are the amount of SO_4_^2–^ and S_tot_, respectively, measured in the same sample.

Fractionation factors (Δ*_*L/H*_*), i.e., in positive per mil (‰) units, were calculated by fitting an approximation of the Rayleigh equation to the data obtained by measuring δ^34^S values of the residual SO_4_^2–^ in the hydroponic solution (δ^34^S_SO_4_^2–^_res_), according to Fry (2006). For these purposes, the following equation was used:


$$\delta^{34}\,\text{S}\,\_\,{\text{SO}}_{4\,\mathrm{res}}^{2-}=\delta^{34}\,\text{S}\,\_\,{\text{SO}}_{4\,\mathrm{source}}^{2-}-\Delta_{L/H}\cdot \text{ln}\,\left(f\right)$$

where *f* is the fraction of SO_4_^2–^ remaining in the hydroponic solution, and δ^34^S_SO_4_^2–^_source_ is the initial S isotope composition of the S source.

Finally, the trajectories of the δ^34^S values of the instantaneous product (S_ist_) that forms, inside the plants, instant by instant in time were calculated using the following equation:


$$\delta^{34}\,\text{S}\,\_\,{\text{S}}_{\text{ist}}=\delta^{34}\,\text{S}\,\_\,{\text{SO}}_{4\,\mathrm{source}}^{2-}-\Delta_{\left(L/H\right)}\cdot \left[1+\ln\left(f\right)\right]$$

### RNA Extraction and Quantitative Real-Time PCR Analysis

Total RNA was extracted from rice roots using TRIzol Reagent (Life Technologies Corporation, Carlsbad, CA, United States) and then purified using PureLink ^®^ RNA Mini Kit (Life Technologies Corporation, Carlsbad, CA, United States), according to the manufacturer’s instructions. Contaminant DNA was removed on-column using PureLink ^®^ DNase (Life Technologies Corporation, Carlsbad, CA, United States). The first-strand cDNA synthesis was carried out using the SuperScript™ III First-Strand Synthesis SuperMix for quantitative real-time PCR (qRT-PCR; Life Technologies Corporation, Carlsbad, CA, United States), according to the manufacturer’s instructions.

The qRT-PCR analysis of *OsSULTR1;1* (LOC_Os03g09970) and *OsSULTR1;2* was performed on the first-strand cDNA in a 20 μl reaction mixture containing GoTaq ^®^ qPCR Master Mix (Promega) and the specific primers, using an ABI 7300 Real-Time PCR system (Applied Biosystems). The relative transcript level of each gene was calculated by the 2^–ΔΔCt^ method using the expression of the *OsS16* (LOC_Os11g03400) gene as reference. Primers for qRT-PCR are listed in Supplementary Table 1.

### Statistical Analysis

Quantitative values are presented as mean ± SEM of three independent experiments run in duplicate (*n* = 3). Two distinct 3-L tanks were used for each condition analyzed in each independent experiment. ANOVA was carried out using SigmaPlot for Windows version 11.0 (SYSTAT Software, Inc., San Jose, CA, United States). The significant values were adjusted for multiple comparisons using the Bonferroni correction. The Student’s *t*-test was used to assess the significance of the observed differences between the values measured in root and shoot.

## Results

### Sulfur Isotope Mass Balance in a Closed Hydroponic-Rice System (Experimental Setup A)

**FIGURE 1:**
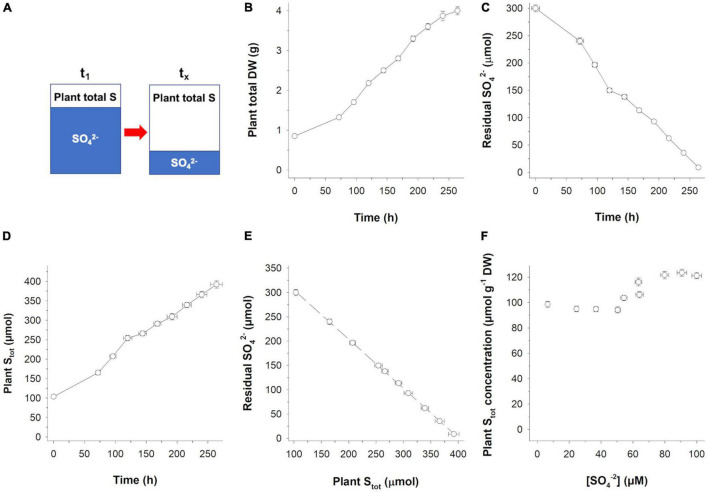
Time course of S partitioning in a closed hydroponic-rice system. **(A)** Experimental setup: A finite amount of sulfate (SO_4_^2–^) is continuously removed from the hydroponic solution and converted into the total S of the plant (S_tot_). **(B)** Plant total biomass accumulation. **(C)** Residual SO_4_^2–^ in the hydroponic solution over time. **(D)** S_tot_ accumulation over time. **(E)** Residual SO_4_^2–^ in the hydroponic solution vs. plant S_tot_ accumulation. **(F)** Plant S_tot_ concentration vs. SO_4_^2–^ concentration in the hydroponic solution. Data are means ± SE of three independent experiments run in duplicate (*n* = 3).

Potential ^32^S/^34^S isotope effects occurring during SO_4_^2–^ uptake were investigated by setting up a closed hydroponic-rice system (Figure 1A) in which a finite amount of substrate (i.e., the SO_4_^2–^ in the nutrient solution) was continuously removed from the solution, by the activity of the SO_4_^2–^ transporters of the roots, and converted into a final product (i.e., S_tot_). Using this system, we performed serial sacrifice experiments in which plant growth was terminated every 24 h (starting from the third day) for the S isotope analyses of both substrates and products.

During the experimental period (264 h), (i) plants continuosly grown (Figure 1B) and removed 98% of the SO_4_^2–^ initially present in the nutrient solution (Figure 1C), (ii) SO_4_^2–^ absorbed was quantitatively recovered in the plants as S_tot_ (Figures 1D,E), and (iii) no significant losses of S occurred during the growth (Figure 1E). The S_tot_ concentration of the plants ranged from 121.2 (at the beginning of the experiment) to 98.6 μmol g^–1^ dry weight (DW; at the end of the experiment), while the SO_4_^2–^ concentration in the nutrient solution ranged from 100 to 6.5 μM, indicating that the regulation of plant S homeostasis occurred during SO_4_^2–^ absorption (Figure 1F).

**FIGURE 2:**
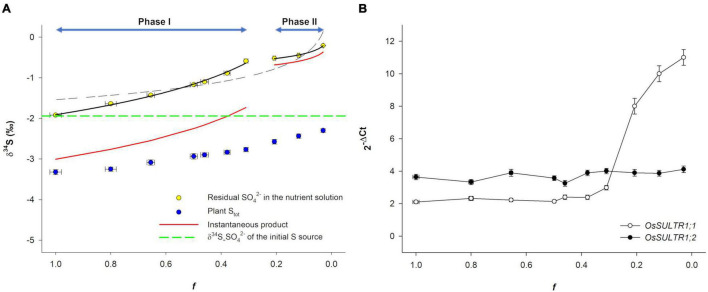
Sulfur isotope dynamic in the closed system and transcriptional analysis of *OsSULTR1;1* and *OsSULTR1;2*. **(A)** S isotope dynamic as a function of the fraction (*f*) of SO_4_^2–^ remaining in the hydroponic solution. Black dashed line is the Rayleigh curve calculated over the entire *f* interval, while the black continuous lines are the Rayleigh curves calculated over the two phases: Phase I (1 ≤ *f* ≤ 0.31) and Phase II (*f* ≤ 0.21). Red continuous lines describe the trajectory of the calculated instantaneous product (S_ist_) over the two phases. The dashed green line indicates the δ^34^S_SO_4_^2–^ value of the initial S source. **(B)** Changes in the relative transcript levels of *OsSULTR1;1* and *OsSULTR1;2* in the roots. The numbers in brackets refer to the concentration of residual SO_4_^2–^ (expressed as μM) in the hydroponic solution corresponding to each *f* value. Data are means ± SE of three independent experiments run in duplicate (*n* = 3).

Figure 2A reports δ^34^S data as a function of the fraction of SO_4_^2–^ remaining in the hydroponic solution (*f*). The δ^34^S of residual SO_4_^2–^ in both the hydroponic solution and plant S_tot_ changed over time, tending toward higher values as *f* decreased. The δ^34^S values of the residual SO_4_^2–^ (δ^34^S_SO_4_^2–^) increased from a minimum of −1.92‰ (at the beginning of the experiment) to a maximum of −0.21‰ (at the final sampling). In contrast, the δ^34^S_S_tot_ of the plants was always lower than the δ^34^S_SO_4_^2–^ of the S source (−1.92 ± 0.02‰) and increased from −3.32‰ (the starting isotope composition of total plant biomass) to −2.30‰ at the final sampling, indicating that SO_4_^2–^ uptake significantly enriches plant S_tot_ in the lighter ^32^S isotope. It is worth noting that, due to mass balance in the closed system, the δ^34^S_S_tot_ of the rice plants tended to the δ^34^S_SO_4_^2–^ of the initial S source as SO_4_^2–^ concentration in the external medium approached zero, indicating that (i) SO_4_^2–^ ions in the nutrient solution were the only S source used by plants and (ii) no significant loses/fractionations of S isotopes occurred during the experiments due to H_2_S gaseous emission (Wilson et al., 1978; Winner et al., 1981).

The isotope effects that occurred in the closed system were analyzed using an approximation of the Rayleigh equation describing isotope partitioning between two reservoirs as one of them decreases in size (Fry, 2006). The S isotope profile of the residual SO_4_^2–^ in the nutrient solution (Figure 2A) showed a marked deviation from a typical Rayleigh enrichment (*R*^2^ = 0.79; black dashed line) due to an unexpected data point distribution at the final steps of the experiment (*f* ≤ 0.21). Considering the Rayleigh fractionation model, it was possible to calculate a single fractionation factor, Δ*_*L/H*_* = 0.48 ± 0.09‰, which describes an average of the net fractionation along the overall trajectory (profile). However, data distribution could be more appropriately described by assuming that a dual-phase Rayleigh fractionation occurred during SO_4_^2–^ uptake. In the first phase (1 ≤ *f* ≤ 0.31), a significant isotope fractionation against ^34^S_SO_4_^2–^ took place (Δ_1_*_(L/H)_* = 1.09‰), while in the second phase (*f* ≤ 0.21), a less pronounced isotope effect [Δ_2_*_(L/H)_* = 0.16‰] was associated with SO_4_^2–^ uptake. Figure 2A also reports the calculated trajectories of the δ^34^S values of S_ist_ that forms, inside the plants, instant by instant in time from the external SO_4_^2–^ due to SO_4_^2–^ uptake; such a product is always offset in the isotope composition of the substrate (δ^34^S_SO_4_^2–^) by the fractionation factor Δ*_*L/H*_* (Fry, 2006). In each phase (I and II), S isotope fractionation (δ^34^S_SO_4_^2–^–δ^34^S_S_ist_) was practically independent of *f*, as can be easily observed by comparing the isotope signatures of the substrate and cumulative product for each data point.

Aiming to decompose the two phases into their physiological and molecular components, we performed a transcriptional analysis of *OsSULTR1;1* and *OsSULTR1;2*, i.e., the main rice genes reasonably involved in SO_4_^2–^ uptake (Godwin et al., 2003; Kumar et al., 2011; Figure 2B). Results revealed that the transition from the two phases was associated with significant changes in the ratio between the transcript levels of the two genes: the *OsSULTR1;2* transcript was always independent of *f* and was more abundant than the *OsSULTR1;1* transcript during the first phase (1 ≤ *f* ≤ 0.31), while the *OsSULTR1;1* transcript level rapidly increased in the second phase (*f* ≤ 0.21), when the SO_4_^2–^ concentration in the nutrient solution became limiting for plant growth [(SO_4_^2–^) ≤ 37 μM; Figure 2B].

### Sulfur Isotope Mass Balance in a Whole Plant: Steady-State vs. Sulfur Starvation (Experimental Setup B)

The possible ^32^S/^34^S isotope effects associated with both S partitioning among plant organs and cell metabolism were investigated by comparing plants pre-grown in complete nutrient solutions and then continuously maintained on media containing SO_4_^2–^ or deprived of SO_4_^2–^ for 72 h (experimental setup B). Nutrient solutions were changed every day to minimize the changes in the isotope signature of the S source (−1.92 ± 0.02‰) due to fractionation associated with SO_4_^2–^ uptake.

**FIGURE 3:**
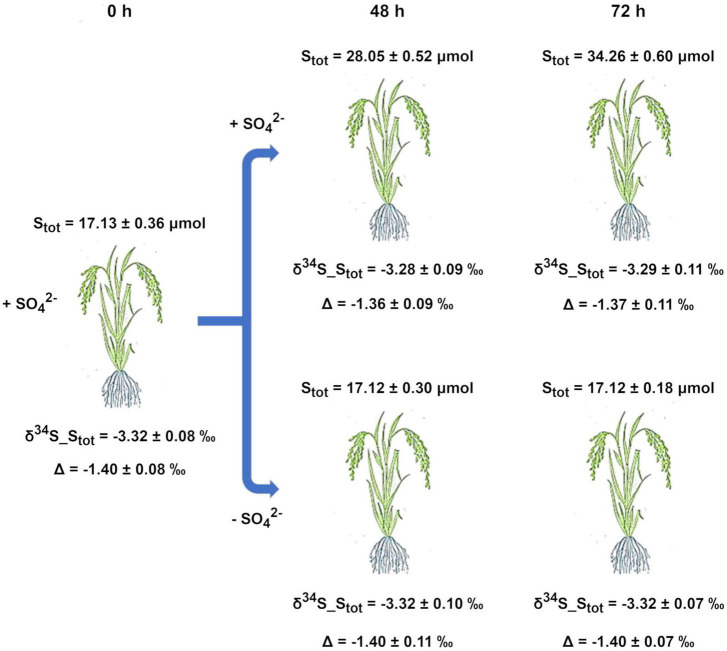
Experimental setup and ^32^S/^34^S isotope effects associated with S partitioning and metabolism in rice plants. Plants were pre-grown in complete nutrient solutions and then continuously maintained on media containing SO_4_^2–^ or deprived of SO_4_^2–^ for 72 h (experimental setup B). S_tot_, total S amount in a whole plant; δ^34^S_S_tot_, S isotope composition of the whole plant; Δ, ^34^S depletion relative to the S source (δ^34^S_SO_4_^2–^_source_ = −1.92 ± 0.02‰). Data are means ± SE of three independent experiments run in duplicate (*n* = 3).

Results showed that the S isotope composition of the whole plants did not significantly change over time since similar δ^34^S_S_tot_ values were measured at each time period (0, 48, and 72 h) in both of the growing conditions (Figure 3). At the beginning of the experiment (0 h), the S_tot_ of the whole plants was significantly depleted in ^34^S by −1.40 ± 0.08‰ relative to the S source (Figure 3).

**FIGURE 4:**
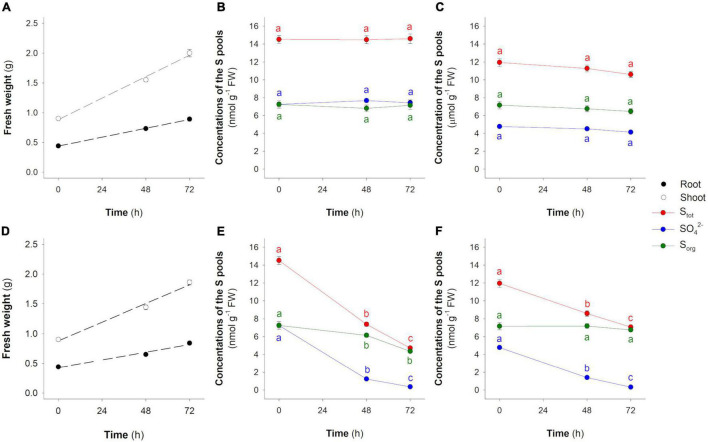
Changes in the concentration of the S pools (S_tot_, SO_4_^2–^, and S_org_) in root and shoot of rice plants grown in the presence or in the absence of SO_4_^2–^ in the hydroponic solution. **(A)** Root and shoot fresh weight (FW) in the presence of SO_4_^2–^. **(B)** S_tot_, SO_4_^2–^, and S_org_ in the root of plants grown in the presence of SO_4_^2–^. **(C)** S_tot_, SO_4_^2–^, and S_org_ in the shoot of plants grown in the presence of SO_4_^2–^. **(D)** Root and shoot FW in the absence of SO_4_^2–^. **(E)** S_tot_, SO_4_^2–^, and S_org_ in the root of plants grown in the absence of SO_4_^2–^. **(F)** S_tot_, SO_4_^2–^, and S_org_ in the shoot of plants grown in the absence of SO_4_^2–^. Data are means ± SE of three independent experiments run in duplicate (*n* = 3). Different letters indicate significant differences between the samples at different times (*P* < 0.05).

**FIGURE 5:**
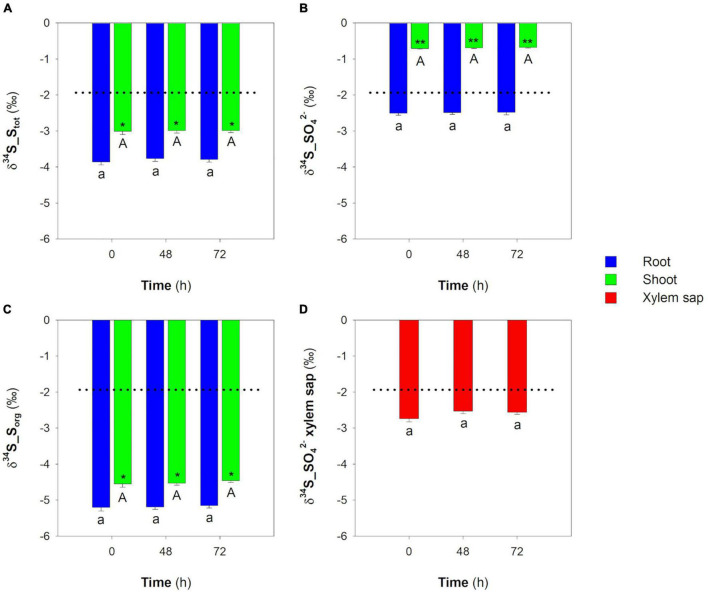
Sulfur isotope composition of the main S pools in root and shoot and of SO_4_^2–^ in the xylem sap of rice plants grown in the presence of SO_4_^2–^ in the hydroponic solution. **(A)** S isotope composition of S_tot_ in root and shoot. **(B)** S isotope composition of SO_4_^2–^ in root and shoot. **(C)** S isotope composition of S_org_ in root and shoot. **(D)** S isotope composition of SO_4_^2–^ in xylem sap. Dotted lines indicate the δ^34^S value of the S source used in the experiment (δ^34^S_SO_4_^2–^_source_ = −1.92 ± 0.02‰). Data are means ± SE of three independent experiments run in duplicate (*n* = 3). Asterisks indicate significant differences (Student’s *t*-test; *0.001 ≤ *P* < 0.05; **P < 0.001) between root and shoot of plants sampled at the same time. Different letters indicate significant differences between the samples (root, shoot, or xylem sap) at different times (*P* < 0.05).

Plants maintained in hydroponic solutions containing SO_4_^2–^ grew linearly in the observation period (Figure 4A). As expected, the concentrations of SO_4_^2–^, S_tot_, and S_org_ did not significantly change in both root and shoot over time (Figures 4B,C). The invariance of each S pool was associated with the invariance of their isotope signatures, indicating that plants reached metabolic and isotope steady-states (Figure 5). The S_tot_ of root and shoot was significantly depleted in ^34^S by −1.94 ± 0.08‰ and −1.09 ± 0.09‰, respectively, relative to the S source (Figure 5A); moreover, δ^34^S_S_tot_ values were significantly lower in the root than in the shoot in all the conditions analyzed (Figure 5A). In the root, the SO_4_^2–^ pools were significantly (*P* < 0.001) ^34^S-depleted relative to the S source, while in the shoot, they were significantly (*P* < 0.001) ^34^S-enriched relative to the same S source (Figure 5B). The S_org_ pool of both root and shoot were significantly ^34^S-depleted with respect to the S source; interestingly, both the S_org_ pools were also significantly ^34^S-depleted with respect to their relative SO_4_^2–^ pools of the cells (Figure 5C). Finally, no differences were found in comparing the δ^34^S_SO_4_^2–^ values of the SO_4_^2–^ pools in the xylem sap and in the whole root system (Figure 5D).

**FIGURE 6:**
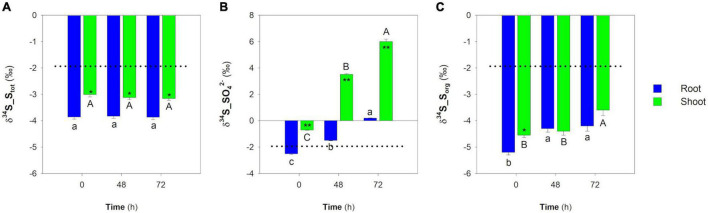
Sulfur isotope composition of the main S pools in root and shoot of rice plants grown in the absence of SO_4_^2–^ in the hydroponic solution. **(A)** S isotope composition of S_tot_ in root and shoot. **(B)** S isotope composition of SO_4_^2–^ in root and shoot. **(C)** S isotope composition of S_org_ in root and shoot. Data are means ± SE of three independent experiments run in duplicate (*n* = 3). Asterisks indicate significant differences (Student’s *t*-test; *0.001 ≤ *P* < 0.05; ** *P* < 0.001) between root and shoot of plants sampled at the same time. Different letters indicate significant differences between the samples (root and shoot) at different times (*P* < 0.05).

In contrast, SO_4_^2–^-deprived plants dynamically allocated S previously absorbed during the preliminary growth phase, preserving both the overall S isotope signature (Figure 3) and the total amount of S_tot_ over time (Figure 4). However, due to both continuous growth (Figure 4D) and S allocation processes, the S_tot_ concentration in rice organs changed over time, decreasing linearly in both root (*R*^2^ = 0.993; Figure 4E) and shoot (*R*^2^ = 0.999; Figure 4F). The SO_4_^2–^ concentration sharply decreased over time in both root and shoot due to SO_4_^2–^ assimilation (Figures 4E,F). In fact, the concentration of the S_org_ in the root slightly decreased over time from 7.26 ± 0.41 to 4.36 ± 0.14 μmol g^–1^ FW, while in the shoot, it remained relatively constant. The S_tot_ isotope composition of both root and shoot did not change over time (Figure 6A) and was significantly ^34^S-depleted relative to the S source. As previously observed, the δ^34^S_S_tot_ values were significantly lower in the root than in the shoot (Figure 6A). Differently, the SO_4_^2–^ pools of both root and shoot became progressively enriched in ^34^S over time. It is worth noting that the most pronounced changes in the SO_4_^2–^ isotope composition were observed in the shoot: the maximum variations observed at 72 h were 2.70 ± 0.05‰ and 6.71 ± 0.19‰ for root and shoot, respectively (Figure 6B). The S_org_ pools of both roots and shoot were significantly ^34^S-depleted compared to the S source; their δ^34^S_S_org_ values changed differently over time since, in the root, they increased moving from 0 to 48 h and then remained constant at 72 h, while in the shoot, a significant increase was observed when moving from 48 to 72 h (Figure 6C).

## Discussion

It is generally assumed that terrestrial plants assimilate S from the soil (SO_4_^2–^) and from the atmosphere (SO_2_) with less S isotope fractionation since the foliar δ^34^S values are generally intermediate between those of the soil and the atmosphere or near to one extreme (Kennedy and Krouse, 1990; Krouse et al., 1991). However, the correct evaluation of the isotope effects due to S acquisition needs a direct comparison between the isotope compositions of the S_tot_ of a whole plant and the S source used by the same plant, since the δ^34^S_S_tot_ value of a single plant organ may result from fractionations and mixing effects occurring during SO_4_^2–^ uptake, assimilation, and partitioning.

To address the S isotope effects during S acquisition, we performed an S isotope mass balance in a closed system, in which the accumulation of S_tot_ in the plants is considered as the result of the continuous consumption of a unique and finite S source (SO_4_^2–^) initially present in a hydroponic solution (Figure 1A). In such a model system, if fractionation occurs, the enrichment in a given isotope in one part of the system results in its depletion in the other, so that isotopic mass balance is always maintained (Fry, 2006).

Our data indicate that isotope discrimination against ^34^S occurred during SO_4_^2–^ uptake, which resulted in transient lighter S isotope compositions of the plants and concomitant ^34^S enrichments of the residual SO_4_^2–^ in the hydroponic solution (Figure 2A). Interestingly, fractionation exhibited two phases characterized by distinct fractionation factors [Δ_1_*_(L/H)_* and Δ_2_*_(L/H)_*] that can be considered as “isotope phenotypes” reflecting plant physiological adaptation to the SO_4_^2–^ concentrations in the nutrient solution, which changed during the experiment (Figures 1F, 2B). The maximum fractionation observed was associated with *f* values ranging from 1 to 0.31 (corresponding to external SO_4_^2–^ concentrations ranging from 100 to 50 μM), while the minimum isotope effect was associated with the smallest *f* values, when the concentration of SO_4_^2–^ in the nutrient solution became critical (≤37 μM) and was potentially able to induce an array of S-deficiency physiological responses (Maruyama-Nakashita et al., 2003), including changes in the expression of the root high-affinity SO_4_^2–^ transports, *OsSULTR1;1* and *OsSULTR1;2*, involved in SO_4_^2–^ uptake (Figure 2B). Although a certain degree of functional redundancy may exist, *OsSULTR1;2* is considered the major gene involved in SO_4_^2–^ uptake under normal conditions, while *OsSULTR1;1* is a more specialized gene that is strongly induced under S limitation (Kumar et al., 2011). The prevalence of *OsSULTR1;1* or *OsSULTR1;2* under different environmental conditions could explain the two isotope phenotypes observed during plant SO_4_^2–^ acquisition, assuming that the different isotope effects may be associated with the activity of the two SO_4_^2–^ transporters. Thus, the plasticity of the isotope phenotype could reflect gene expression in response to changes in both environmental conditions and plant S-nutritional status.

Less information is currently available about the ^32^S/^34^S isotope effects occurring during S partitioning and metabolism in plants since the cycling of the S pools in a whole plant may attenuate the isotope differences between organs potentially caused by S reduction and assimilation. Most of the SO_4_^2–^ ions that are taken up by root are translocated to the shoot, where they are assimilated into organic compounds (Takahashi et al., 2011). However, part of SO_4_^2–^ is also assimilated into the root, and the continuous exchanges of SO_4_^2–^ and S_org_ compounds occur in a shoot-to-root direction in order to ensure the S homeostasis of the root (Cooper and Clarkson, 1989; Bell et al., 1995; Yoshimoto et al., 2003; Larsson et al., 2006).

To analyze the isotope effects occurring during S partitioning and metabolism, we carried out experiments aimed at the following: (i) preventing possible perturbations due to the continuous changes of the δ^34^S_SO_4_^2–^ values of the external solution and (ii) obtaining rice plants with the same overall S isotope composition (Figure 3). In these experiments, plants can be considered systems continuously supplied by an S source that does not change in concentration and isotope composition.

As previously described, plants continuously grown in the presence of SO_4_^2–^ reached metabolic and S isotope steady-states characterized by the invariance of the concentration and the isotope signature of each S pool. It should be noted that the S isotope distribution between root and shoot observed in this study strongly differs from the pattern described by Tcherkez and Tea (2013), concerning the S natural isotope composition in different organs (roots, leaf, stem, glumes, and grains) of mature wheat. Such a discrepancy might depend on the growth conditions (closed hydroponic-plant system vs. field) or, more likely, on the different S nutritional status and/or growth stage of the plants considered in the two studies since S stable isotope separations conserve the memory of the physiological and metabolic activities that determined them. Finally, differences may also originate from the different distribution of the S assimilation enzymes in the leaf, since rice assimilates SO_4_^2–^ mainly in the bundle sheaths, while other species, also in the mesophyll (Hua et al., 2021).

The isotope composition of the SO_4_^2–^ pools of the root was lighter relative to the S source but heavier with respect to the expected composition calculated according to the isotope discrimination occurring during SO_4_^2–^ uptake at high external concentrations [i.e., δ^34^S_SO_4_^2–^ > δ^34^S_SO_4_^2–^_source_–Δ_1_*_(L/H)_*]. Interestingly, SO_4_^2–^ translocation from root to shoot did not discriminate the S isotopes since no differences were found when comparing the isotope signatures of the SO_4_^2–^ ions in root and xylem sap (Figure 5). However, the SO_4_^2–^ pools of the shoot were significantly ^34^S-enriched with respect to the SO_4_^2–^ pools of both root and xylem sap. This was likely due to SO_4_^2–^ assimilation that, favoring the lighter ^32^S isotope, causes a ^34^S enrichment of the residual SO_4_^2–^ ions left behind. The occurrence of an S isotope separation during SO_4_^2–^ assimilation is consistent with the observation that the S_org_ pools of the shoot were significantly depleted in ^34^S relative to both the SO_4_^2–^ pools of the shoot and the S source. Since the aerial portion of the plant is fed by the SO_4_^2–^ ions continuously translocated from root to shoot and the S_tot_ of the shoot was lighter relative to the SO_4_^2–^ coming from the root, we can reasonably suppose that a non-negligible portion of the ^34^S-enriched SO_4_^2–^ of the shoot is translocated to the root. Thus, the isotope signature of the SO_4_^2–^ pool of the root could be the result of mixing effects due to the overall S isotope circulation, SO_4_^2–^ uptake, and local S assimilation. Assuming that during the S isotope steady-state, (i) the δ^34^S_SO_4_^2–^ values measured in the root are mainly influenced by root SO_4_^2–^ uptake and SO_4_^2–^ translocation from shoot to root, and (ii) the S isotope composition of the instantaneous SO_4_^2–^ that continuously enters the root cells should theoretically differ from the S source by the fractionation factor Δ_1_*_(L/H)_*, so that


$$\delta^{34}\,\text{S}\,\_\,{\text{SO}}_{4\,\mathrm{ist}}^{2-}=\delta^{34}\,\text{S}\,\_\,{\text{SO}}_{4\,\mathrm{source}}^{2-}-\Delta_{1\,\left(L/H\right)}=-1.92\,‰-1.09\,\text{‰}=-3.01\,\text{‰}$$

We can estimate the maximum amount of SO_4_^2–^ that, coming from the shoot, is translocated and accumulated into the root (defined as SO_4_^2–^_StoR_) by imposing the following mass balance:


$$\delta^{34}\,\text{S}\,\_\,{\text{SO}}_{4\,\mathrm{root}}^{2-}\cdot {\text{SO}}_{4\,\mathrm{root}}^{2-}=\delta^{34}\,\text{S}\,\_\,{\text{SO}}_{4\,\mathrm{shoot}}^{2-}\cdot {\text{SO}}_{4\,\mathrm{StoR}}^{2-}+\delta^{34}\,\text{S}\,\_\,{\text{SO}}_{4\,\mathrm{ist}}^{2-}\cdot \left({\text{SO}}_{4\,\mathrm{root}}^{2-}-{\text{SO}}_{4\,\mathrm{StoR}}^{2-}\right)$$

where δ^34^S_SO_4_^2–^_root_ is the steady-state isotope composition of the SO_4_^2–^ pool of the root, SO_4_^2–^_root_ is the total amount of the SO_4_^2–^ measured in the root, and δ^34^S_SO_4_^2–^_shoot_ is the isotope composition of the SO_4_^2–^ ions coming from the shoot. Solving the equation for the unknown SO_4_^2–^_StoR_ reveals that, in our conditions, 21.7% of the steady-state SO_4_^2–^ pool of the rice root is inherited from the shoot.

Although less information is currently available on the long-distance transport of SO_4_^2–^ from shoot to root, we can reasonably suppose that such an activity may involve the phloem and specific isoforms of SO_4_^2–^ transporters mediating the loading of SO_4_^2–^ into the sieve tubes (Takahashi, 2019). Feeding experiments with ^35^SO_4_^2–^ performed on *Arabidopsis* (Yoshimoto et al., 2003) support our finding, indicating the retranslocation of SO_4_^2–^ as an important activity in controlling root SO_4_^2–^ homeostasis and S isotope composition.

In contrast, during the growing period in the absence of SO_4_^2–^, rice plants can be considered closed systems assimilating the SO_4_^2–^ ions previously absorbed during the preliminary growth phase and allocating the S_org_ pools to optimize the distribution of the limited S resources between root and shoot. It is worth noting that in these conditions, the invariance of the S_tot_ isotope composition of both root and shoot was associated with dramatic changes in the isotope composition of the relative SO_4_^2–^ and S_org_ pools (Figure 6), mainly caused by the ^32^S/^34^S isotope effects occurring during SO_4_^2–^ assimilation. During the observation period, plants rapidly consumed the available SO_4_^2–^ pools: at the end of the experiment, the overall S_org_ pool was about 94% of the S_tot_. The S isotope mass balance that was carried out considering the overall SO_4_^2–^ and S_org_ pools of the plants (i.e., root + shoot; Table 1) revealed that continuous S assimilation progressively enriched both the overall S_org_ pool in the lighter ^32^S isotope and the residual SO_4_^2–^ in the heavier ^34^S isotope, producing an apparent isotope separation that was closely dependent on the severity of the imposed S starvation, as indicated by calculated Δ values (Δ = δ^34^S_S_org_−δ^34^S_SO_4_^2–^) that ranged from −3.29 ± 0.40 (at the beginning of the experiment) to −7.80 ± 0.18‰ (at 72 h). As expected, the most pronounced isotope separations were observed in the shoot, confirming the prominent role of the rice aerial portion in SO_4_^2–^ assimilation and S allocation (Takahashi et al., 2011).

**TABLE 1:** Amount and sulfur (S) isotope composition of the overall SO_4_^2–^ and S organic (Sorg) pools of rice plants grown in the absence of SO_4_^2–^ in the hydroponic solution.

	Time (h)					
	0		48		72	
	Amount (μmol)	δ^34^S (‰)	Amount (μmol)	δ^34^S (‰)	Amount (μmol)	δ^34^S (‰)
SO_4_^2–^	7.48 ± 0.13^a^	−1.47 ± 0.05^c^	2.80 ± 0.05^b^	2.07 ± 0.08^b^	0.90 ± 0.02^c^	4.06 ± 0.18^a^
S_org_	9.65 ± 0.38^c^	−4.76 ± 0.28^a^	14.32 ± 0.31^b^	−4.73 ± 0.17^a^	16.22 ± 0.18^a^	−3.73 ± 0.17^b^

*Data are means ± SE of three independent experiments run in duplicate (n = 3). Different letters indicate significant differences between the samples at different times (P < 0.05).*

## Conclusion and Perspectives

Our results provide an overview of the ^32^S/^34^S isotope effects occurring during SO_4_^2–^ uptake, partitioning, and metabolism in rice. The main results clearly show that SO_4_^2–^ uptake discriminates against ^34^S, enriching plant total biomass in the lighter ^32^S isotope relative to the S source. The S isotope discrimination observed during SO_4_^2–^ acquisition closely depends on the amount of SO_4_^2–^ in the growing medium, as well as on the plants’ molecular and physiological responses aimed at optimizing S nutrition under different environmental conditions. Although further experiments will be necessary to directly measure the isotope effect associated with the activity of a single SO_4_^2–^ transporter, we can reasonably conclude that *OsSULTR1;1* and *OsSULTR1;2* differently discriminate against ^34^S, producing S isotope phenotypes closely dependent on their relative expression.

Results also indicate that the steady-state S isotope composition of the different S pools of both root and shoot mainly results from the substantial S isotope fractionations occurring during SO_4_^2–^ assimilation and mixing effects due to the overall isotope circulation inside the whole plant. Finally, the extreme variability of the S isotope phenotypes observed under various S conditions underlines the potential of the δ^34^S analysis to provide information for further detailed characterization of the metabolic and molecular processes involved in plant S homeostasis, as well as of the plant S systemic fluxes occurring in different nutritional and environmental conditions, since the S stable isotope separations conserve the memory of the activities that determined them.

## Data Availability Statement

The raw data supporting the conclusions of this article will be made available by the authors, without undue reservation.

## Author Contributions

VC, MC, GS, and FN designed the experiments. VC, MM, and MC performed the experiments. FN analyzed the data and wrote the manuscript. All authors revised the manuscript draft and approved the final version.

## Conflict of Interest

The authors declare that the research was conducted in the absence of any commercial or financial relationships that could be construed as a potential conflict of interest.

## Publisher’s Note

All claims expressed in this article are solely those of the authors and do not necessarily represent those of their affiliated organizations, or those of the publisher, the editors and the reviewers. Any product that may be evaluated in this article, or claim that may be made by its manufacturer, is not guaranteed or endorsed by the publisher.

## References

[B1] Bell C. I. Clarkson D. T. Cram W. J. (1995). Partitioning and redistribution of sulphur during S-stress in Macroptilium atropurpureum cv. Siratro. *J. Exp. Bot.* 46 73–81.

[B2] Blair G. J. Till A. R. (2003). *Guidelines for the Use of Isotopes of Sulfur in Soil-plant Studies.* Vienna: International Atomic Energy Agency (IAEA).

[B3] Buchner P. Takahashi H. Hawkesford M. J. (2004). Plant sulphate transporters: co-ordination of uptake, intracellular and long-distance transport. *J. Exp. Bot.* 55 1765–1773. 10.1093/jxb/erh206 15258169

[B4] Cooper H. D. Clarkson D. T. (1989). Cycling of amino-nitrogen and other nutrients between shoots and roots in cereals – a possible mechanism integrating shoot and root in the regulation of nutrient uptake. *J. Exp. Bot.* 40 753–762. 10.1093/jxb/40.7.753 12432039

[B5] Coplen T. B. Krouse H. R. (1998). Sulphur isotope data consistency improved. *Nature* 392:32.

[B6] De Laeter J. R. Böhlke J. K. De Bièvre P. Hidaka H. Peiser H. S. Rosman K. J. R. (2003). Atomic weights of the elements: review 2000 (IUPAC technical report). *Pure Appl. Chem.* 75 683–800.

[B7] Epstein E. (2000). “The discovery of the essential elements” in *Discoveries in Plant Biology.* eds Kung S.-D. Yang S.-F. (Singapore: World Scientific Publishing). 1–16. 10.1142/9789812813503_0001

[B8] Fry B. (2006). *Stable Isotope Ecology.* New York: Springer.

[B9] Gigolashvili T. Kopriva S. (2014). Transporters in plant sulfur metabolism. *Front. Plant Sci.* 5:442. 10.3389/fpls.2014.00442 25250037 PMC4158793

[B10] Godwin R. M. Rae A. L. Carrol B. J. Smith F. W. (2003). Cloning and characterization of two genes encoding sulfate transporters from rice (Oryza sativa L.). *Plant Soil* 257 113–123. 10.1023/a:1026202709134

[B11] Günal S. Hardman R. Kopriva S. Mueller J. W. (2019). Sulfation pathways from red to green. *J. Biol. Chem.* 294 12293–12312. 10.1074/jbc.REV119.007422 31270211 PMC6699852

[B12] Hua L. Stevenson S. R. Reyna-Llorens I. Xiong H. Kopriva S. Hibberd J. M. (2021). The bundle sheath of rice is conditioned to play an active role in water transport as well as sulfur assimilation and jasmonic acid synthesis. *Plant Cell* 107 268–286. 10.1111/tpj.15292 33901336

[B13] Kemp A. L. W. Thode H. G. (1968). The mechanism of the bacterial reduction of sulphate and of sulphite from isotope fractionation studies. *Geochim. Cosmochim. Acta* 32 71–91.

[B14] Kennedy B. V. Krouse H. R. (1990). Isotope fractionation by plants and animals: implications for nutrition research. *Can. J. Physiol. Pharmacol.* 68 960–972. 10.1139/y90-146 2200589

[B15] Krouse H. R. Stewart J. W. B. Grinenko V. A. (1991). “Pedosphere and biosphere” in *Stable Isotopes: Natural and Anthropogenic Sulfur in the Environment, SCOPE 43.* eds Krouse H. R. Grinenko V. A. (New York: John Wiley & Sons). 267–306.

[B16] Kumar S. Asif M. A. Chakrabarty D. Tripathi R. D. Trivedi P. K. (2011). Differential expression and alternative splicing of rice sulphate transporter family members regulate sulphur status during plant growth, development and stress conditions. *Funct. Integr. Genomics* 11 259–273. 10.1007/s10142-010-0207-y 21221698

[B17] Larsson C. M. Larsson M. Purves J. V. Clarkson D. T. (2006). Translocation and cycling through roots of recently absorbed nitrogen and sulfur in wheat (Triticum aestivum) during vegetative and generative growth. *Physiol. Plant.* 82 345–352.

[B18] Leustek T. Martin M. N. Bick J. A. Davies J. P. (2000). Pathways and regulation of sulfur metabolism revealed through molecular and genetic studies. *Annu. Rev. Plant. Physiol. Plant. Mol. Biol.* 51 141–165. 10.1146/annurev.arplant.51.1.141 15012189

[B19] Maghrebi M. Baldoni E. Lucchini G. Vigani G. Valè G. Sacchi G. A. (2021). Analyisis of cadmium root retention for two contrasting rice accessions suggests an important role for OsHMA2. *Plants* 10:806. 10.3390/plants10040806 33923918 PMC8073749

[B20] Maruyama-Nakashita A. Inoue E. Watanabe-Takahashi A. Yamaya T. Takahashi H. (2003). Transcriptome profiling of sulfur-responsive genes in Arabidopsis reveals global effects of sulfur nutrition on multiple metabolic pathways. *Plant Physiol.* 132 597–605. 10.1104/pp.102.019802 12805590 PMC167000

[B21] Mekhtiyeva V. L. (1971). Isotopic composition of sulfur of plants and animals from reservoirs of various salinity. *Geokhimiya* 6 725–730.

[B22] Rees C. E. (1973). A steady-state model for sulphur isotope fractionation in bacterial reduction processes. *Geochim. Cosmochim. Acta* 37 1141–1162. 10.1016/0016-7037(73)90052-5

[B23] Sacchi G. A. Nocito F. F. (2019). Plant sulfate transporters in the low phytic acid network: some educated guesses. *Plants* 8:616. 10.3390/plants8120616 31861241 PMC6963184

[B24] Sachs J. (1865). *Handbuch der Experimental-Physiologie der Pflanzen.* Leipzig: Wilhelm Engelmann.

[B25] Saito K. (2004). Sulfur assimilatory metabolism. The long and smelling road. *Plant Physiol.* 136 2443–2450. 10.1104/pp.104.046755 15375200 PMC523311

[B26] Tabatabai M. A. Bremner J. M. (1970). A simple turbidimetric method of determining total sulfur in plant material. *Agron. J.* 62 805–806.

[B27] Takahashi H. (2019). Sulfate transport systems in plants: functional diversity and molecular mechanisms underlying regulatory coordination. *J. Exp. Bot.* 70 4075–4087. 10.1093/jxb/erz132 30907420

[B28] Takahashi H. Kopriva S. Giordano M. Saito K. Hell R. (2011). Sulfur assimilation in photosynthetic organisms: molecular functions and regulations of transporters and assimilatory enzymes. *Annu. Rev. Plant Biol.* 62 157–184. 10.1146/annurev-arplant-042110-103921 21370978

[B29] Tcherkez G. Tea I. (2013). 32S/34S isotope fractionation in plant sulphur metabolism. *New Phytol.* 200 44–53. 10.1111/nph.12314

[B30] Thode H. G. Macnamara J. Collins C. B. (1949). Natural variations in the isotopic content of sulphur and their significance. *Can. J. Res.* 27 361–373. 10.1139/cjr49b-038 18130453

[B31] Trust B. A. Fry B. (1992). Stable sulphur isotopes in plants: a review. *Plant Cell Environ.* 15 1105–1110. 10.1111/j.1365-3040.1992.tb01661.x

[B32] Wilson L. G. Bressan R. A. Filner P. (1978). Light-dependent emission of hydrogen sulfide from plants. *Plant Physiol.* 61 184–189. 10.1104/pp.61.2.184 16660257 PMC1091829

[B33] Winner W. E. Smith C. L. Koch G. W. Mooney H. A. Bewley J. D. Krouse H. R. (1981). Rates of emission of H2S from plants and patterns of stable sulphur isotope fractionation. *Nature* 289 672–673.

[B34] Yoshimoto N. Inoue E. Saito K. Yamaya T. Takahashi H. (2003). Phloem-localizing sulfate transporter, Sultr1;3, mediates re-distribution of sulfur from source to sink organs in Arabidopsis. *Plant Physiol.* 131 1511–1517. 10.1104/pp.014712 12692311 PMC166910

