# Supplementary material for: Sulfur Stable Isotope Discrimination in Rice: A Sulfur Isotope Mass Balance Study
Source: Front Plant Sci. 2022 Mar 10;13:837517. doi: 10.3389/fpls.2022.837517 (PMC8960986; doi:10.3389/fpls.2022.837517)
Supplement: Supplementary file 1 [file Data_Sheet_1.pdf]

Supplementary Material

Sulfur Stable Isotope Discrimination in Rice: a Sulfur Isotope Mass Balance Study

Viviana Cavallaro[1], Moez Maghrebi[1,2], Mariachiara Caschetto[1,3], Gian Attilio Sacchi[1], Fabio Francesco Nocito[1*]

[1]Dipartimento di Scienze Agrarie e Ambientali – Produzione, Territorio, Agroenergia, Università degli Studi di Milano, Milano, Italy

[2]Dipartimento di Scienze della Vita e Biologia dei Sistemi, Università degli Studi di Torino, Torino, Italy

[3]Dipartimento di Scienze dell'Ambiente e della Terra, Università degli Studi di Milano-Bicocca, Milano, Italy

*** Correspondence:**
Fabio Francesco Nocito
fabio.nocito@unimi.it

Table S1. Primers used for qRT-PCR analysis.

**Gene**	**Primer name**	**Sequence**
OsSULTR1;1	OsSULTR1;1-F	GGAGCATTCTTTGGCGTCAT
	OsSULTR1;1-R	TCGCAACCGCAATTAGCA
OsSULTR1;2	OsSULTR1;2-F	CGACCTTCTTTGCAGGAGTCA
	OsSULTR1;2-R	TGAACCCTAGCCTGCAGAAAC
OsS16	OsS16-F	ACGTCGACGAGGCATCCA
	OsS16-R	CGCGACCACCGAACTTCTT

Table S2. S isotope composition of the main S pools in root and shoot and of SO_4^{2-} in the xylem sap of rice plants grown in the presence of SO_4^{2-} in the hydroponic solution.

	Sample	Time (h)					
		0		48		72	
		$\delta^{34}S$ (‰)	Δ (‰)	$\delta^{34}S$ (‰)	Δ (‰)	$\delta^{34}S$ (‰)	Δ (‰)
S_{tot}	Root	-3.86 ± 0.08 [a]	-1.94 ± 0.08 [a]	-3.76 ± 0.09 [a]	-1.84 ± 0.09 [a]	-3.79 ± 0.08 [a]	-1.87 ± 0.08 [a]
	Shoot	-3.01 ± 0.09 [a*]	-1.09 ± 0.09 [a*]	-2.99 ± 0.07 [a*]	-1.07 ± 0.08 [a*]	-2.99 ± 0.06 [a*]	-1.07 ± 0.07 [a*]
SO_4^{2-}	Root	-2.51 ± 0.05 [a]	-0.59 ± 0.06 [a]	-2.49 ± 0.05 [a]	-0.57 ± 0.05 [a]	-2.48 ± 0.07 [a]	-0.56 ± 0.08 [a]
	Shoot	-0.71 ± 0.02 [a**]	1.21 ± 0.03 [a**]	-0.69 ± 0.02 [a**]	1.23 ± 0.03 [a**]	-0.68 ± 0.02 [a**]	1.24 ± 0.03 [a**]
S_{org}	Root	-5.20 ± 0.10 [a]	-3.28 ± 0.10 [a]	-5.19 ± 0.07 [a]	-3.27 ± 0.07 [a]	-5.15 ± 0.07 [a]	-3.23 ± 0.07 [a]
	Shoot	-4.55 ± 0.09 [a*]	-2.63 ± 0.09 [a*]	-4.53 ± 0.06 [a*]	-2.61 ± 0.06 [a*]	-4.46 ± 0.05 [a*]	-2.54 ± 0.06 [a**]
SO_4^{2-}	Xylem sap	-2.47 ± 0.09 [a]	-0.55 ± 0.09[a]	-2.53 ± 0.07 [a]	-0.61 ± 0.07[a]	-2.56 ± 0.06 [a]	-0.64 ± 0.06[a]

Δ indicates changes in ^{34}S relative to the S source ($\delta^{34}S$-$SO_4^{2-}{}_{source}$ = -1.92 ± 0.02 ‰). Data are means and SE of three independent experiments run in duplicate (n = 3). Asterisks indicate significant differences (Student's *t*-test; * $0.001 \leq P < 0.05$; ** $P < 0.001$) between root and shoot of plants sampled at the same time. Different letters indicate significant differences among the samples (root, shoot, or xylem sap) at different times ($P < 0.05$).
